# Microglia response and function in a chronic model of photoreceptor damage

**DOI:** 10.3389/fcell.2025.1699271

**Published:** 2025-12-16

**Authors:** Deepa Raghavan, Olivia Jeakle, Yasmeen Berry, Majd Victor, Ryan Thummel

**Affiliations:** Department of Ophthalmology, Visual and Anatomical Sciences, Wayne State University School of Medicine, Detroit, MI, United States

**Keywords:** microglia, photoreceptor, degeneration, zebrafish, Muller glia

## Abstract

**Background:**

Retinal neurodegenerative diseases, including diabetic retinopathy and age-related macular degeneration, are characterized by the slow, chronic degeneration of photoreceptors. We previously used a chronic low light (CLL) exposure to model slow photoreceptor degeneration in adult zebrafish. Here, we investigate transcriptional, morphological, and functional responses of microglia in the CLL model.

**Methods:**

Microglia-specific gene expression analysis was mined from our previously reported 3′ RNA-seq data performed at 8 time points during 28 days of CLL exposure. Morphological changes were performed on retinas collected at various time points using immunohistochemistry. Microglial inhibition was accomplished pharmacologically with dexamethasone and genetically using the *irf8−/−* mutant fish. Finally, we returned the CLL-treated fish to normal light/dark conditions to test whether photoreceptors could recover in the context of chronic stress.

**Results:**

CLL induced dynamic, time-dependent upregulation of microglia-specific genes consistent with pro-inflammatory and pro-resolving function. Dexamethasone treatment reduced microglial numbers and exacerbated rod and cone outer segment damage, whereas *irf8−/−* mutants exhibited partial protection against photoreceptor damage. Notably, despite prolonged stress and damage during the CLL exposure, photoreceptor outer segments returned to near-baseline morphology after 28 days of normal light/dark recovery conditions.

**Discussion:**

Overall, these findings suggest that microglial function in chronic retinal injury is context-dependent as pharmacological and genetic methods of inhibition produced contrasting outcomes depending upon microglial polarization.

## 1 Introduction

The leading cause of adult vision impairment and blindness worldwide is a retinal neurodegenerative disease known as diabetic retinopathy (Ali et al., 2020; Antonetti et al., 2021). Along with other chronic retinal diseases such as age-related macular degeneration and glaucoma, this condition involves a slow and progressive photoreceptor dysfunction that eventually leads to vision impairment or complete vision loss (Ali et al., 2020; Mitchell et al., 2018b; Saadane et al., 2021). Recent research using mammalian vision models has resulted in some promising outcomes (Palazzo et al., 2022; Todd et al., 2021; Todd and Reh, 2022); however, the limited ability of mammals to replace lost retinal neurons is a significant obstacle. To explore alternative model organisms for studying the genetic and molecular mechanisms underlying retinal damage and repair, many research workers in retinal cell biology have turned to zebrafish. In addition to their advantages such as large brood size, ease of maintenance, and availability of genetic manipulation tools (Lenkowski and Raymond, 2014), zebrafish possess the unique ability to endogenously restore vision following extensive and acute retinal damage (Lenkowski and Raymond, 2014; Vihtelic and Hyde, 2000).

Many acute damage paradigms have been developed in zebrafish, including intense phototoxic lesion, neurotoxins, lasers, and physical damage (Fausett and Goldman, 2006; Harvey et al., 2019; Jahnke and Enzmann, 2011; Montgomery et al., 2010; Nagashima et al., 2013; Rumford et al., 2024; Zhang et al., 2016). However, the most common method to induce retinal damage in adult zebrafish is acute high-intensity light (AL). This process selectively destroys outer rod and cone photoreceptors while sparing the inner retina (Kumar et al., 2025). Studies using this and other models have shown that acute retinal cell death in zebrafish leads to the activation of the resident retinal macroglia, Müller glia (MG) (Bernardos et al., 2007; Fausett and Goldman, 2006; Fimbel et al., 2007; Lenkowski and Raymond, 2014; Thummel et al., 2008). Zebrafish MG undergo asymmetric cell division to generate a Müller glia-derived progenitor cell (MGPC) that, in turn, amplifies to produce cells, ultimately differentiating into new photoreceptors (Nagashima et al., 2013).

In our previous work, we characterized a 28-day regeneration time course following AL-induced photoreceptor destruction. We combined morphological and gene expression analyses to better understand the dynamic process of zebrafish retinal regeneration (Kramer et al., 2021). Although these studies have revealed nuanced cellular and genetic events that occur in response to acute damage, they do not accurately reflect the slow photoreceptor degeneration observed in most human retinal diseases. To address this limitation, chronic retinal degeneration models have been developed. Two of the first models described in zebrafish were a XOPS-mCFP transgenic line that caused selective rod degeneration and a null mutation in the *pde6c* gene that caused cone degeneration (Morris et al., 2008; Morris et al., 2005). Both models demonstrated photoreceptor regeneration in response to damage as early as 7 days post-fertilization, providing a system to study chronic damage from early retinal development. In adult zebrafish, the *bbs2*−/− line has been used to model the chronic disease Bardet–Beidl syndrome (BBS) (Song et al., 2020). In this model, cone photoreceptor degeneration triggered a robust inflammatory response, with significantly more microglia observed in the outer retina at 7 months post-fertilization compared to wild-type controls (Song et al., 2020). However, the chronic damage was insufficient to induce MG cell cycle re-entry, apparently remaining below the “threshold” required to trigger a robust regenerative response. Finally, we developed a photoreceptor degeneration model induced by chronic low light (CLL) exposure (Turkalj et al., 2021), including a thorough characterization of gene expression patterns and glial cell morphology during a 28-day photoreceptor degeneration time course (Kramer et al., 2023). Like the XOPS-mCFP and *bbs2−/−* chronic models, we found that despite significant truncation of the photoreceptor outer segments, CLL-induced damage remained below the threshold for MG proliferation (Kramer et al., 2023; Turkalj et al., 2021).

Recent studies have examined the role of microglia in both acute and chronic damage contexts, often through pharmacological or genetic inhibition. For example, targeted immunomodulation of microglia in zebrafish larval retinas was reported using the glucocorticoid dexamethasone (Dex) (White et al., 2017). Interestingly, Dex-induced immune suppression co-administered with ablative injury delayed MG proliferation, whereas Dex treatment after cell loss accelerated regeneration (White et al., 2017). Another method of microglial inhibition was described using the genetic mutant *irf8* ^*st95*^, which carries a null allele of *irf8* caused by a frameshift deletion (Song et al., 2024). As Irf8 is a master inflammatory transcription factor and is critical for microglia and other macrophage development, *irf8* null mutants lack microglia at early larval stages (Shiau et al., 2015). Interestingly, adult *irf8* ^*st95*^ mutants displayed normal photoreceptor regeneration following AL injury, suggesting that microglia may not be essential in the acute context and could be compensated for by alternative mechanisms (Song et al., 2024).

This raises the question of whether microglia play a functional role in the context of chronic damage, where persistent photoreceptor degeneration and prolonged stress may depend on sustained immune–glial interactions. In our previous work, we observed that microglia with morphology consistent with intermediate activation reside at the ROS/RPE margin, even prior to phototoxic damage (Kramer et al., 2023). Interestingly, during CLL, these microglia increase in number and remain at the outer edge of the rod outer segment (ROS) even as the outer segments slowly truncate and degenerate toward the inner retina (Kramer et al., 2023). In this work, we utilized both pharmacological (Dex) and genetic (*irf8*) microglial inhibition in CLL treatment to better understand the nuanced role of microglia in modulating photoreceptor degeneration. We also showed that photoreceptor outer segments are fully restored following a return of CLL-treated fish to normal light/dark conditions, despite enduring 28 days of chronic stress.

## 2 Materials and methods

### 2.1 Husbandry

All experiments were performed using adult *albino* (*alb*) zebrafish aged 6–9 months. Fish were maintained in a closed water circulation system. Unless otherwise noted, fish were maintained in a standard circadian light cycle of 14 h of light (∼250 lux) and 10 h of dark at 28.5 °C and fed 3× daily with a combination of brine shrimp and flake food (Westerfield, 2000). All procedures used in this study were approved by the Institutional Animal Care and Use Committee (IACUC) at Wayne State University (Protocol # 25-01-7472).

### 2.2 Light treatment protocol

Prior to CLL treatment, fish were placed in a 24-h dark adaptation. CLL followed previously established protocols and utilized a 28-day treatment course (Kramer et al., 2021; Turkalj et al., 2021). In brief, CLL exposed 24 fish to two 250W halogen light bulbs continuously for 28 days followed by a 28-day recovery period. For each tissue collection, N = 5–8 animals were euthanized, and whole eyes were harvested. Only one eye per animal was utilized for analysis. Eyes were collected at four time points: 1) at the cessation of CLL treatment at 28 days post-light onset (28dpl), 2) 28dpl + 7-day recovery, 3) 28dpl + 14-day recovery, and 4) 28dpl + 28-day recovery. Two control groups were collected at the time of the final tissue collection: 1) 24-h dark-adapted controls (0 h) and 2) naïve controls from standard light–dark conditions (ctrl).

### 2.3 Systemic immunosuppression

As previously described (Kyritsis et al., 2012), we used dexamethasone (Dex; Sigma, D1756), a synthetic glucocorticoid, to achieve systemic immunosuppression. A stock solution was prepared with 25 mg Dex per 1 mL 0.6% methanol (MeOH). A volume of 600 μL of stock solution was added to 1 L tank water to obtain 15 mg/L Dex. An equal volume of 0.6% methanol was added to tank water for the MeOH carrier control fish. Fish were immersed in 15 mg/L Dex, 0.06% MeOH (carrier control), or untreated system water (control) for 13 consecutive days under standard light/dark conditions (14 h light:10 h dark), followed by a 24-h dark adaptation. Tank water was exchanged daily, and fish were fed with GEMMA 300 once every other day, at least 3 h prior to water exchange. Immediately after dark adaptation, the control fish (N = 8) were euthanized, and eyes were collected, while the Dex and MeOH fish began CLL (∼6,000 lux). After 5 consecutive days of CLL exposure, eyes were collected from Dex (N = 7) and MeOH (N = 8) fish.

### 2.4 Immunohistochemistry

Fish were euthanized in a 1:500 solution of 2-phenoxyethanol. Eyes were collected from euthanized fish and placed in a 9:1 ethanolic formaldehyde solution overnight at 4 °C. Eyes were then washed 2× in 5% sucrose/1X phosphate-buffered saline (PBS) at room temperature (RT) for 30 min and 1 h, respectively. After the second wash, eyes were placed in 30% sucrose/1XPBS overnight at 4 °C. The next day, eyes were placed in a 1:1 solution of 30% sucrose/1XPBS and Tissue Freezing Medium (TFM; General Data, Cincinnati, OH) and incubated overnight at 4 °C. Eyes were then embedded in 100% TFM and stored at −80 °C. Embedded eyes were cryosectioned at a thickness of 16 μm, and sections were collected on Superfrost Plus glass microscope slides (Fisher Scientific, Waltham, MA). Slides were dried on a slide warmer at 55 °C for 1–2 h prior to storage at −80 °C.

Stored slides were re-dried for 20 min on a slide warmer at 55 °C, after which the tissue was rehydrated with 1XPBS for 20 min at RT. Slides were then incubated in blocking solution (2% normal goat serum, 0.2% Triton X-100, and 1% dimethyl sulfoxide in 1XPBS) for 1 h at RT and subsequently incubated with primary antibodies diluted in blocking solution overnight at RT. The following day, slides were washed 3× for 10 min with 0.05% Tween-20 in 1XPBS (0.05% 1XPBT). Secondary antibodies and TO-PRO-3 (Life Technologies, Grand Island, NY), a nuclear stain, were diluted 1:500 in 0.05% 1XPBT. Slides were incubated in diluted secondary antibodies and TO-PRO-3 for 1 h at RT, after which the tissue was again washed 3× for 10 min with 0.05% 1XPBT. Slides were then mounted for microscopy using ProLong Gold Antifade Reagent (Molecular Probes, Eugene, OR).

The following primary antibodies were used: mouse anti-4c4 (gift from Peter Hitchcock; 1:250), mouse anti-zpr-3 (Zebrafish International Resource Center; 1:200), mouse anti-zpr-1 (Zebrafish International Resource Center; 1:200), rabbit anti-red opsin (gift from David Hyde; 1:500), rabbit anti-green opsin (gift from David Hyde; 1:1,000), rabbit anti-blue opsin (gift from David Hyde; 1:500), rabbit anti-UV opsin (gift from David Hyde; 1:1,000), and mouse anti-PCNA (Sigma; 1:1,000). Secondary antibodies used were all AlexaFluor-conjugated 488 and 594 anti-primary (Life Technologies, Grand Island, NY; 1:500).

### 2.5 TUNEL analysis

A terminal deoxynucleotidyl transferase dUTP nick end labeling (TUNEL) assay was used to detect DNA damage as a proxy for cells undergoing apoptosis. Stored slides were re-dried for 20 min on a slide warmer at 55 °C and subsequently rehydrated in 1XPBS for 20 min at RT. Following rehydration, slides were incubated in ApoTag® Equilibration Buffer (EMD Millipore, Darmstadt, Germany) for 10 min at 37 °C. The TUNEL reaction mix was prepared according to the manufacturer’s protocol, using TdT dNTP mix (R&D Systems, Minneapolis, MN) and TdT enzyme (Takara Bio, Japan, Korea). Slides were incubated in the TUNEL reaction mix for 2 h at 37 °C. Slides were then washed with 2X SSC buffer for 15 min at RT, followed by two washes with 1XPBS. Streptavidin-conjugated AlexaFluor 488 (Life Technologies, Grand Island, NY; 1:200), which was used to detect incorporated biotinylated dNTPs, and TO-PRO-3 (1:500) were diluted in a solution of 0.1% Tween-20 in 1XPBS (0.1% 1XPBT). Slides were incubated in the Streptavidin solution for 1 h at RT and then washed 3× with 0.1% 1XPBT. After a final wash in 1XPBS, the slides were mounted for microscopy using ProLong Gold Antifade Reagent.

### 2.6 Confocal microscopy and image quantification

Using a Leica TCS SP8 confocal microscope, all images from a given experiment were acquired from the central dorsal retina on a single plane with the same exposure and gain using a ×20 lens (NA = 0.75) and a ×1.5 optical zoom. Images were processed using Fiji (ImageJ) for quantification of both mean fluorescence intensity and photoreceptor outer segment lengths. For mean fluorescence intensity, the fluorescence channel of interest was isolated, and mean fluorescence intensity within a given region of interest was measured (N = 5–8 images from each treatment group quantified per marker). Fluorescent intensities were normalized to the control baseline intensity. For outer segment lengths, 10 measurements were taken across the linear length of each image and were averaged. The full thickness of the rod outer segment layer was measured to avoid inaccurate measurements due to difficulty delineating individual rod outer segments. Given the minimal overlapping of cone outer segments, individual cone outer segments were identified and measured. Length measurements were taken in arbitrary units and converted to μm. For the 4c4 staining of microglia and TUNEL assay, cells were hand-counted over a 300-μm linear distance in the central dorsal retina. 4c4^+^ microglia were counted in four regions of the retina: ganglion cell layer (GCL), inner plexiform and nuclear layers (IPL + INL), outer plexiform and nuclear layers (OPL + ONL), and outer segments and subretinal space (OS + SRS). In all cases, the users were blinded to the experimental groups they were quantifying.

To validate manual counts of microglia, an automated image processing pipeline was also created using CellProfiler 4.2.8 (CellProfiler Image Analysis Software, RRID:SCR_007358), as previously conducted (Kramer et al., 2023). The same RGB confocal images as used in manual counts were separated into their individual channel components using ImageJ 1.53k (Schneider et al., 2012). For each image, a region of interest was manually defined based on the TO-PRO-3 signal and applied as a mask to all corresponding channel images for further analysis. The green channel images were converted to gray scale, and the 4c4 fluorescence signal was enhanced using the *EnhanceOrSuppressFeatures* module with the neurite feature type and line structure enhancement method, feature size of 25. Cell bodies were identified from the enhanced 4c4 grayscale images as primary objects using an adaptive robust background thresholding approach, with a typical object diameter ranging from 15 to 75 pixels. The lower outlier fraction was set to 0.05, the upper outlier fraction was set to 0.05, and a mean averaging method with two standard deviations was used. A threshold smoothing scale of 1.3488, a correction factor of 1.5, threshold bounds of 0.0–1.0, and an adaptive window size of 50 were applied. Object areas were measured in pixels with the *MeasureObjectSizeShape* module, and the resulting cell counts and area were exported to a spreadsheet.

A CellProfiler pipeline was also used to quantitatively analyze PCNA + cells, utilizing methods adapted from a pipeline published in previous work (Berry et al., 2025). Images prepared through the TO-PRO-3 nuclear stain blue channel were converted to gray scale, and the fluorescence signal was enhanced using the *CorrectIlluminationCalculate* and *CorrectIlluminationApply* modules. For each image, a region of interest was manually defined as the outer nuclear layer (ONL), and the test mode was used to initially optimize settings for the identification of the objects of interest. PCNA + cells used the *IdentifyPrimaryObjects* module with global thresholding based on the Otsu method (Otsu, 1979), applying a two-class thresholding approach. The typical diameter of objects was set to a range from 10 to 35 pixels, with objects outside this range discarded. A smoothing scale of 1.3488 and a correction factor of 0.20 were applied. Threshold bounds were between 0.0 and 1.0. Clumped objects were distinguished based on intensity, and dividing lines were drawn based on shape. After objects were identified, the resulting cell counts were exported to a spreadsheet.

### 2.7 Statistical analysis

For the 4c4^+^ cell counts, a two-way ANOVA was used to determine statistical significance across treatment groups and retinal layers, followed by a *post hoc* Tukey test to determine statistical differences between treatment groups and retinal layers. For all other quantification methods, a one-way ANOVA was performed to determine statistical significance across treatment groups or the time course, followed by a *post hoc* Tukey test to determine statistical differences between treatment groups or time points. Statistical analyses were completed using GraphPad Prism 10.

### 2.8 Microglial gene expression analysis

#### 2.8.1 Gene selection

We first compiled a list of 99 genes associated with microglia based on genes identified from previously reported scRNA-seq data (Iribarne and Hyde, 2022; Lyu et al., 2023; Mazzolini et al., 2019). We then used 3′mRNA-seq (Quantseq) data previously generated in our laboratory (Kramer et al., 2023) to obtain transcript-level pseudocounts for each gene of interest at various time points following the CLL treatment (Turkalj et al., 2021). Data were obtained from seven time points, which included 0 h post-light (hpl), 24 hpl, 72 hpl, 5 days post-light (dpl), 10 dpl, 14 dpl, and 28 dpl. Note that we eliminated the 36 hpl time point from analysis due to variations observed at this time point that were likely driven by circadian rhythm. For each gene, the average of n = 6 replicates was calculated at each time point, resulting in a single transcript level for each of the seven time points. Next, to narrow the list of microglial genes of interest, we eliminated several genes based on the following criteria.

1. Genes that were not found in previous 3′mRNA-seq data
2. Genes with a peak transcript level < 20
3. Genes with stable transcript levels throughout the 28-day time course

After this elimination process, we were left with a list of 17 microglial genes of interest with fluctuating transcript levels over the 28-day time course. GraphPad Prism 10 was used to plot transcript levels over time for each of the 17 genes.

#### 2.8.2 k-means cluster analysis

For each gene, the log_2_fold change in transcript levels from 0 hpl at each time point was calculated as follows: log_2_ (transcript level at 0 hpl − transcript level at 0 hpl), log_2_ (transcript level at 24 hpl − transcript level at 0 hpl), log_2_ (transcript level at 72 hpl − transcript level at 0 hpl), etc. The dataset containing log_2_fold change values was imported into RStudio version 2024.09.0 + 375 to perform the k-means cluster analysis. To determine the optimal number of clusters, a within-sum-of-squares plot was created in RStudio. The “elbow” point of the plot fell at 6, indicating that k = 6 was the optimal number of clusters. The k-means cluster analysis was performed using k = 6 centers, 10 as the maximum number of interactions, and 25 as the number of random starting partitions.

## 3 Results

### 3.1 Transcriptional analysis of microglial-specific genes during chronic low light damage revealed dynamic changes associated with microglia activation

**FIGURE 1:**
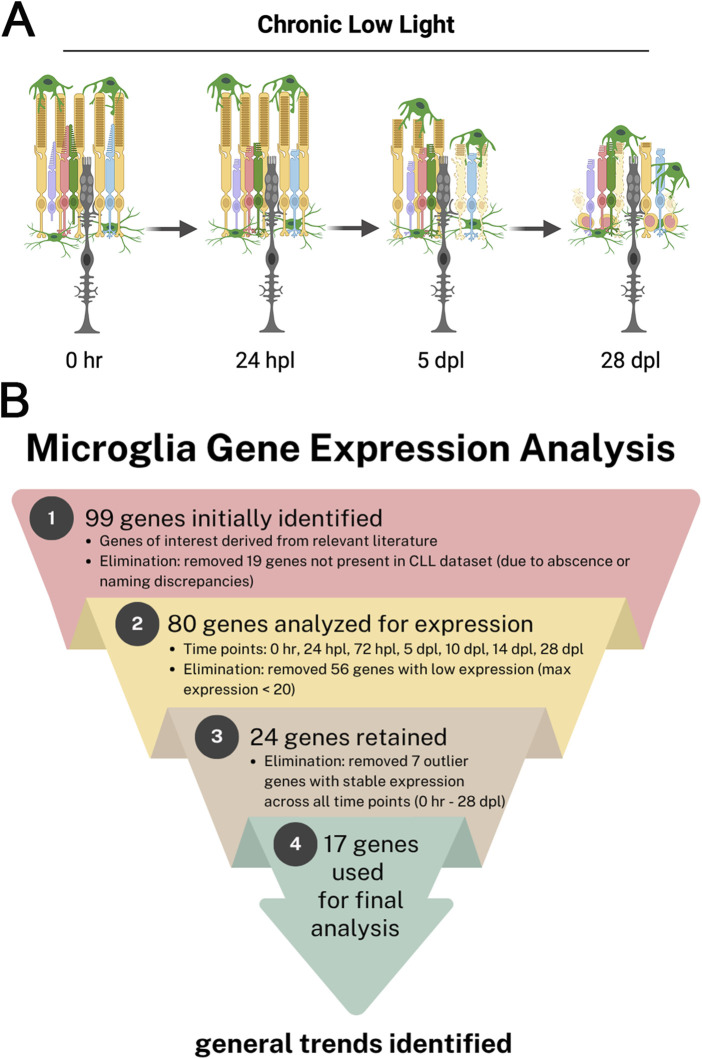
Microglia response to a chronic low intensity light (CLL) model of photoreceptor injury in adult zebrafish. **(A)** Schematic representation of the CLL damage model and the subsequent changes to microglia, rod, and cone photoreceptors over the 28-day time course. As rod photoreceptor outer segments (ROSs) were truncated over the course of the light treatment, microglia in the outermost retina remained in contact with the distal tips of the ROS. Morphologically, microglia in the outermost retina appeared to remain in an intermediate state of activation throughout the time course, whereas microglia in the outer plexiform layer remained in a ramified/resting state: created in BioRender.com (2025) https://BioRender.com/ju6zoeu. **(B)** Microglia gene expression analysis workflow from initial genes to general trends identified. A total of 99 genes were identified from relevant literature (Iribarne and Hyde, 2022; Lyu et al., 2023; Mazzolini et al., 2019), and subsequent stages of elimination resulted in 17 genes used for final analysis.

We previously characterized a 28-day CLL photoreceptor damage model in adult zebrafish, including morphological and transcriptional analyses at eight key time points, from 0 h to 28 days post-light onset (dpl) (Kramer et al., 2023). There, we described differential responses of microglia in the CLL model *versus* the more commonly used intense acute light model. In particular, even in undamaged retinas, we showed that microglia in the ROS/sub-retinal space exhibited a rounded morphology, representing an intermediate activation state (Kramer et al., 2023). This contrasted with the microglia in the plexiform layers, which exhibited a ramified/resting morphology (Kramer et al., 2023). Additionally, during CLL, microglia in the outermost retina remained in close contact with the slowly degenerating ROS throughout the time course, forming a near contiguous row of microglia at the RPE/ROS interface (Figure 1A) (Kramer et al., 2023).

To better understand the microglial-specific transcriptional changes that occur alongside this observation, we searched our 3′RNA-seq database for genes previously identified in the literature by scRNA-seq as microglial-specific (Iribarne and Hyde, 2022; Lyu et al., 2023; Mazzolini et al., 2019). Of the 99 genes initially identified from the relevant literature, 80 were present in our 3′RNA-seq database (Figure 1). These genes were subsequently analyzed for expression across multiple time points over the course of the CLL treatment: 0 h, 24 hpl, 72 hpl, 5 dpl, 14 dpl, and 28 dpl. Following this analysis, 56 of the genes were eliminated from subsequent analysis due to low transcript levels. Of the 24 retained genes, 7 exhibited stable expression across all time points (Supplementary Figure S1). The remaining 17 genes were utilized in our final analysis to identify general trends (Figure 1B).

**FIGURE 2:**
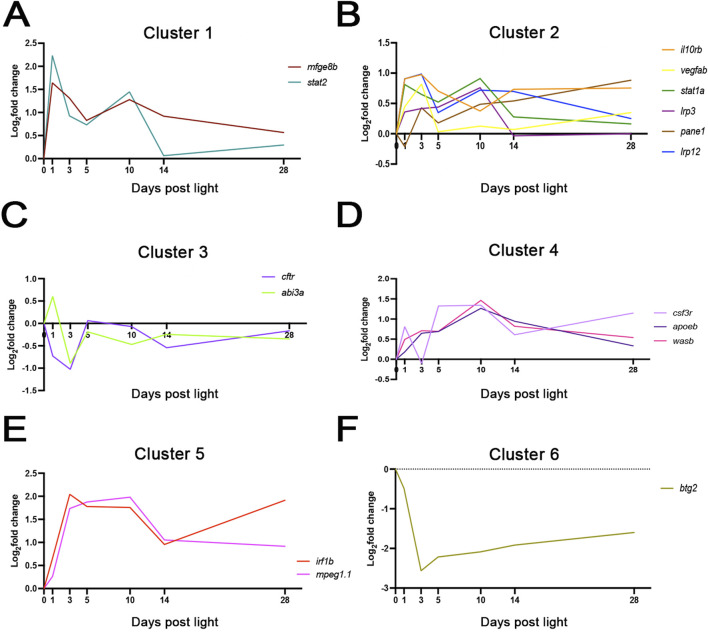
k-means clustering revealed dynamic gene expression changes associated with microglia activation. k-means clustering analysis was performed on the 17 genes of interest across the 28-day CLL time course. Clusters 1 and 2 **(A,B)** clustered genes that showed a strong upregulation at 24–72 h post-light exposure (hpL). Cluster 3 **(C)** grouped two genes with a down regulation by 72 hpL. Cluster 4 **(D)** grouped three genes with a peak of expression between 5 and 10 dpl. Cluster 5 **(E)** clustered two genes with a peak upregulation beginning at 24 hpl and sustained through 10 dpl. Finally, Cluster 6 **(F)** showed only one gene with an expression peak at 0 h, followed by a precipitate drop and sustained downregulation.

We first manually clustered the genes into groups based on the following: an early rise and peak trend between 24 hpl and 72 hpl (Supplementary Figure S2), a peak at 5 dpl (Supplementary Figure S3A,B), a peak at 10 dpl (Supplementary Figure S3C–F), a peak at 28 dpl (Supplementary Figure S3G), and a peak at 0 h followed by a downregulation throughout the time course (Supplementary Figure S3H). To validate findings from this manual curation, an unbiased k-means clustering analysis was conducted, which revealed six clusters that largely matched our manual clustering (Figure 2). Of note, both showed that 16 of the 17 transcripts were upregulated in gene expression levels (Figures 2A–E), with only *btg2* showing sustained downregulation (Figure 2F). Collectively, these findings suggest a transcriptional activation of microglia that is associated with the slow degeneration of photoreceptors in the CLL model.

### 3.2 Dexamethasone-mediated microglia inhibition resulted in reduced microglia number and accelerated rod and cone outer segment damage

**FIGURE 3:**
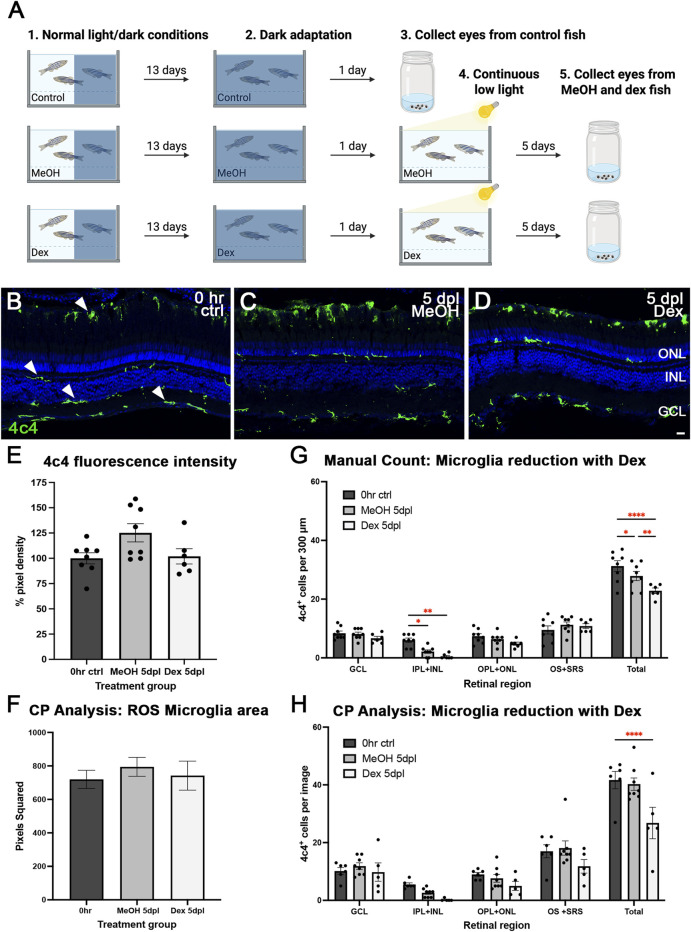
Dexamethasone (Dex) treatment reduced the total number of microglia. **(A)** Graphic depiction of the methodology used in this study. Control, untreated system water; MeOH, 0.06% methanol carrier control; Dex, 15 mg/L dexamethasone: created in BioRender.com (2025) https://BioRender.com/ykzrpzd. **(B)** Control, **(C)** MeOH 5dpl, **(D)** and Dex-treated 5dpl retinas stained with 4c4 (green) to label microglia. TO-PRO-3 (blue) was used to stain nuclei. **(E)** We observed no significant difference in 4c4 fluorescence intensity (p = 0.1152) between MeOH and Dex. **(F)** A CellProfiler analysis was used to quantitively assess the ROS microglial area and found no statistically significant difference between conditions. **(G)** The total number of microglia did not decrease in the GCL, OPL + ONL, or OS + SRS under MeOH and Dex-treated conditions. However, the total number of microglia was reduced by 10% (p = 0.0443) with MeOH and 27% (p < 0.0001) with Dex compared to 0 h ctrl. This reduction stemmed primarily from the IPL + INL region, where the microglia number was reduced by 78% (p = 0.0173) in MeOH and 92% (p = 0.0012) in Dex compared to 0 h ctrl. **(H)** CellProfiler analysis was used to validate manual counts and found a 36% reduction in the total number of microglia between 0 h ctrl and Dex 5 dpl (p < 0.0001). N = 6–8 eyes analyzed per condition (values are mean ± SEM; scale bar in panel F = 15 µm).

Given that we found transcriptional data suggestive of microglia activation across the CLL time course (Figures 2A–E; Supplementary Figure S2A–I; Supplementary Figure S3A–G), we aimed to test the role of microglia, if any, in rod and cone outer segment damage. Dex, a glucocorticoid receptor agonist, has been previously shown to pharmacologically inhibit the function of microglia in the retina (Emmerich et al., 2023). Based on previous findings (Kyritsis et al., 2012), we exposed zebrafish to 15 mg/L Dex, 0.06% methanol (MeOH; carrier control), or untreated system water (control) for 13 days under a standard light/dark cycle (14 h light:10 h dark) (Figure 3A). Following 24 h of dark adaptation, eyes were collected from control fish (0 h ctrl), whereas MeOH- and Dex-treated fish were additionally exposed to CLL for 5 additional days (Figure 3A). After eye collection, immunohistochemistry was performed, and microglia were immunolabeled with a 4c4 marker, while nuclei were stained with TO-PRO-3 (Figures 3B–D). We observed no difference in 4c4 fluorescence intensity (p = 0.1152) between MeOH and Dex (Figure 3E). A CellProfiler analysis was also used to quantitatively assess the ROS microglial area, and no statistically significant difference was observed between conditions (Figure 3F). However, after 5 days of CLL, the total number of microglia was reduced by 10% (p = 0.0443) in the MeOH group and 27% (p < 0.0001; Figure 3G) in the Dex-treated group, when compared to 0 h ctrl. There was also a significant difference between the MeOH group and the Dex-treated animals (p = 0.0032). Interestingly, this decrease was largely due to a loss of ramified/resting microglia in the inner plexiform layer (IPL) and inner nuclear layer (INL), where the microglial number was reduced by 78% (p = 0.0173) in MeOH and 92% (p = 0.0012) in Dex relative to 0 h ctrl. In contrast, no significant changes in microglia were observed in the GCL, OPL + ONL, or OS + SRS (Figure 3G). CellProfiler analysis was used to validate manual cell counts and found similar trends, with a 36% reduction in the total number of microglia between 0 h ctrl and Dex 5 dpl (p < 0.0001; Figure 3H). Together, these results suggest that Dex reduces microglial number, particularly in the inner retina.

**FIGURE 4:**
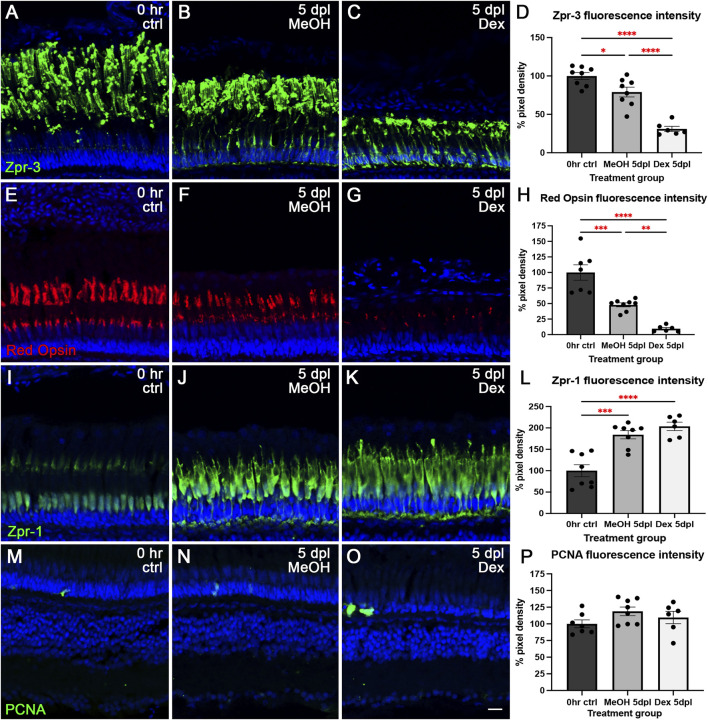
ROS and cone photoreceptor damage was exacerbated following Dex treatment. Control **(A)**, MeOH 5dpl **(B)**, and Dex-treated 5dpl retinas **(C)** stained with Zpr-3 (green) to label ROS. TO-PRO-3 (blue) was used to stain nuclei. **(D)** Compared with controls, there was a 21% (p < 0.05) reduction in Zpr-3 fluorescence intensity with MeOH and a 69% (p < 0.0001) reduction with Dex treatment. Control **(E)**, MeOH 5dpl **(F)**, and Dex-treated 5dpl retinas **(G)** stained with Red Opsin (red) to label cone photoreceptors. **(H)** Compared with controls, Red Opsin fluorescence intensity decreased by 52% (p = 0.0002) with MeOH and by 90% (p < 0.0001) with Dex treatment. Control **(I)**, MeOH 5dpl **(J)**, and Dex-treated 5dpl retinas **(K)** stained with Zpr-1/Arrestin3a (green) to label red–green double cones. **(L)** Compared with controls, Zpr-1 fluorescence intensity increased by 84% (p = 0.0001) with MeOH and doubled (p < 0.0001) with Dex treatment. Control **(M)**, MeOH 5dpl **(N)**, and Dex-treated 5dpl retinas **(O)** stained with PCNA to label proliferating cells. **(P)** There was no significant difference in PCNA fluorescence intensity between MeOH and Dex (p = 0.6468). N = 6–8 eyes analyzed per condition (values are mean ± SEM; scale bar in panel K = 15 µm).

To test whether Dex treatment results in any microglia-mediated phenotypic outcomes, we next labeled 0 h, MeOH-, and Dex-treated retinas with markers for rod and cone outer segments, proliferation, and cell death. We used Zpr-3 to immunolabel the ROS (Figures 4A–C) and found an exacerbation of damage in both MeOH- and Dex-treated animals relative to controls. In particular, we observed a 21% (p < 0.05) reduction in Zpr-3 fluorescence intensity in the MeOH group and a 69% (p < 0.0001) reduction in the Dex-treated group at 5 dpl (Figure 4D). Importantly, there was also a significant difference between the MeOH group and the Dex-treated animals (p < 0.0001). This corresponded a 12-fold (p = 0.321) increase in photoreceptor apoptosis of rod photoreceptor nuclei in the ONL with MeOH and 36-fold (p < 0.0001) increase with Dex treatment relative to controls (Supplementary Figure S4). Next, we showed that Red Opsin fluorescence intensity in red/green double cone photoreceptors decreased 52% (p = 0.0002) in the MeOH group and 90% (p < 0.0001) with Dex treatment (Figures 4E–H). Again, we also observed a significant difference between the MeOH group and the Dex-treated animals (p = 0.006). Interestingly, unlike rod photoreceptor loss in the Dex-treated animals, immunolabeling of the cell somas with Zpr-1 (i.e., Arrestin 3a) revealed that cone photoreceptors are still present in the Dex-treated group, despite the loss of their COS (Figures 4I–L). Finally, despite the greater extent of outer segment damage and rod photoreceptor apoptosis observed in the Dex group, we failed to observe Müller glial proliferation (Figures 4M–O), which is a hallmark of acute damage paradigms (Kumar et al., 2025). In addition, there was no significant difference in PCNA + fluorescence intensity from the sporadic proliferation in rod precursor cells in the ONL between MeOH and Dex (p = 0.6468; Figure 4P). Together, these data suggest that microglia are largely neuroprotective in the CLL and that pharmacological inhibition of their function resulted in exacerbated damage to photoreceptors.

### 3.3 *irf8−/−* animals exhibited partial protection against rod and cone photoreceptor damage during CLL treatment

**FIGURE 5:**
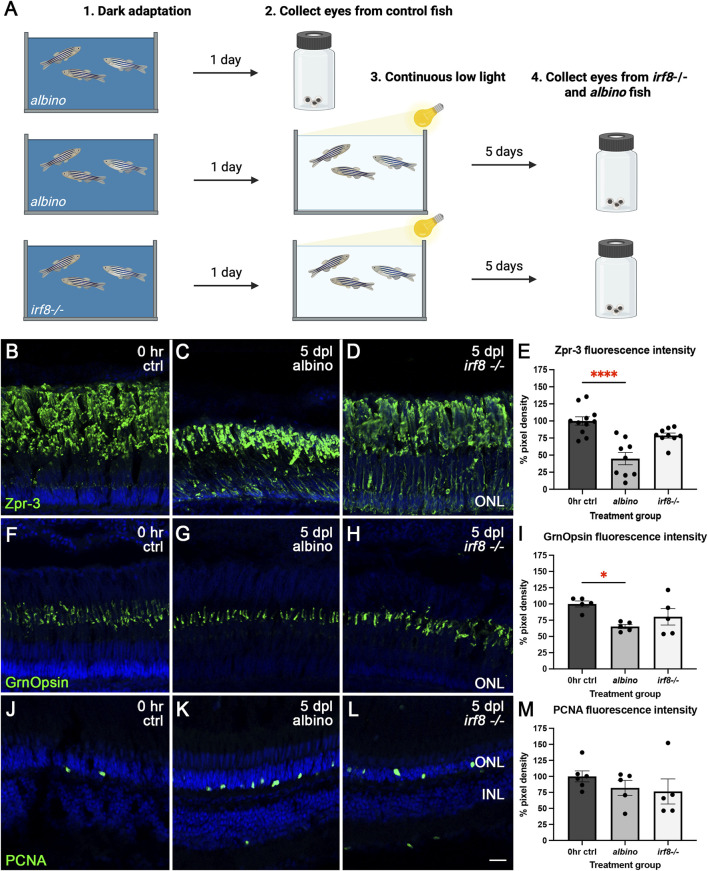
ROS and cone photoreceptor damage was partially rescued by genetic microglia inhibition in *irf8−/−*;*albino* zebrafish. **(A)** Graphic depiction of the methodology used in this study: created in BioRender.com (2025) https://BioRender.com/x0i0li8. Control **(B)**, *albino* 5dpl **(C)**, and *irf8−/−*;*albino* 5dpl **(D)** retinas stained with Zpr-3 (green) to label the ROS. TO-PRO-3 (blue) was used to stain nuclei. **(E)** Compared with controls, there was a 55% (p < 0.001) reduction in Zpr-3 fluorescence intensity in *albino* fish. Although Zpr-3 fluorescence intensity for the *irf8−/−*;*albino* fish decreased by an average of 21%, the difference was not statistically significant (p = 0.0520). Control **(F)**, *albino* 5dpl **(G)**, and *irf8−/−*;*albino* 5dpl **(H)** retinas stained with GrnOpsin (green) to label the cone photoreceptors. **(I)** Compared with controls, there was a 35% (p = 0.0184) reduction in GrnOpsin fluorescence intensity with *albino*, whereas no significant difference was observed in *irf8−/−*;*albino* fish (p = 0.1857). Control **(J)**, *albino* 5dpl **(K)**, and *irf8−/−*;*albino* 5dpl **(L)** retinas stained with PCNA (green) to label proliferating cells. **(M)** There was no significant difference in PCNA fluorescence intensity in *albino* (p = 0.5548) and *irf8−/−;albino* (p = 0.3825) compared to controls. N = 4–5 eyes analyzed per condition (values are mean ± SEM; scale bar in panel K = 15 µm).

To complement our pharmacological approach, we also used a genetic model of microglia loss to test whether the reduction in the microglia number also resulted in exacerbated damage to photoreceptors in the CLL model. *irf8−/−;albino* mutants display significantly depleted numbers of microglia (Song et al., 2024). Mimicking the Dex paradigm, following 24 h of dark adaptation, eyes were collected from control *albino* fish (0 h ctrl), *albino* fish at 5 dpl, and *irf8−/−;albino* at 5 dpl (Figure 5A). Immunohistochemistry was performed using Zpr-3 to label the ROS, Green Opsin to label the COS of red/green double-cones, and PCNA to monitor proliferation. We found that relative to controls, there was a 55% (p < 0.001) reduction in Zpr-3 fluorescence intensity in *albino* fish (Figures 5B,C,E). However, the slight decrease observed in Zpr-3 fluorescence intensity for the *irf8−/−;albino* fish was not statistically significant (p = 0.0520; Figures 5C–E), suggesting that *irf8−/−;albino* fish were more resistant to ROS damage in the CLL model. Similarly, we observed a 35% (p = 0.0184) reduction in Green Opsin fluorescence intensity in *albino* animals relative to controls (Figures 5F,G,I) and no significant difference in *irf8−/−;albino* fish (p = 0.1857; Figures 5G–I). Collectively, these findings differ from our previously described pharmacological Dex inhibition, which instead exacerbated the reduction. The only similar finding was that genetic microglial inhibition did not stimulate significant Müller glial proliferation (Figures 5J–M); we observed no sign of Müller glial proliferation and no significant difference in PCNA fluorescence intensity from rod precursors in the ONL in *albino* (p = 0.5548) and *irf8−/−;albino* (p = 0.3825; Figure 5M).

### 3.4 Following CLL damage, return to normal light/dark conditions resulted in recovery of rod and cone outer segments

**FIGURE 6:**
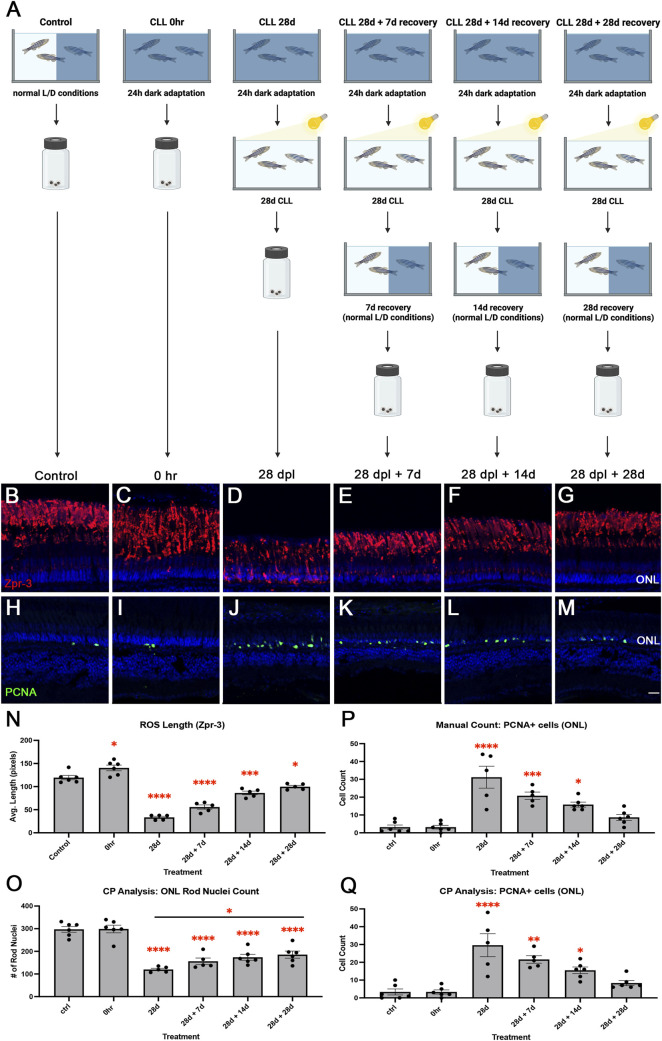
Gradual restoration in ROS length and decrease in ONL proliferation was observed during a recovery period following CLL treatment. **(A)** Graphic depiction of the methodology used in this study: created in BioRender.com (2025) https://BioRender.com/l6wg0cg. Retinas were stained with Zpr-3 (red; **(B–G)** to label ROS and with PCNA (green; **(H–M)**) to label proliferating cells. TO-PRO-3 (blue) was used to stain nuclei. **(B,H)** control. **(C,I)** 0 h. **(D,J)** 28 dpl. **(E,K)** 28 dpl + 7 days of recovery. **(F,L)** 28 dpl +14 days of recovery. **(G,M)** 28 dpl + 28 days of recovery. **(N–Q)** Graphical representations of quantitative data for ROS and PCNA. Asterisks indicate significantly different than control. **(N)** 24 h dark adaptation (0 h) resulted in a modest but significant increase in ROS length compared to control (p = 0.0203). By the end of CLL treatment (28d), the ROS length was reduced by 72% (p < 0.0001). Following a return to normal light/dark conditions, ROS length began to recover to 53% (p < 0.0001) after 7 days of recovery, 28% (p = 0.0003) after 14 days of recovery, and 16% (p = 0.0492) by 28 days of recovery. **(O)** CellProfiler (CP) analysis was used to quantitatively assess ONL rod nuclei count. By the end of CLL treatment (28d), the ONL rod nuclei count was reduced by 60% (p < 0.001) compared to control. Counts gradually increased to 47% less (p < 0.001) by 7 days of recovery, 42% (p < 0.001) by 14 days of recovery, and 38% (p < 0.001) by 28 days of recovery. There was a 55% increase from 28d to 28d + 28d (*p < 0.05). **(P)** PCNA + cells in the ONL showed near 10-fold (p < 0.0001) increase compared to baseline at 28 dpl. This was followed by gradual decreases at 7 days of recovery (p = 0.0007), 14 days of recovery (p = 0.0150), and resolution by 28 days of recovery (p = 0.6362). **(Q)** Validation of manual count using CellProfiler showed similar trends (****p < 0.001, **p = 0.0011, and *p = 0.0360). N = 4–6 eyes analyzed per condition (values are mean ± SEM; scale bar in panel M = 15 µm).

**FIGURE 7:**
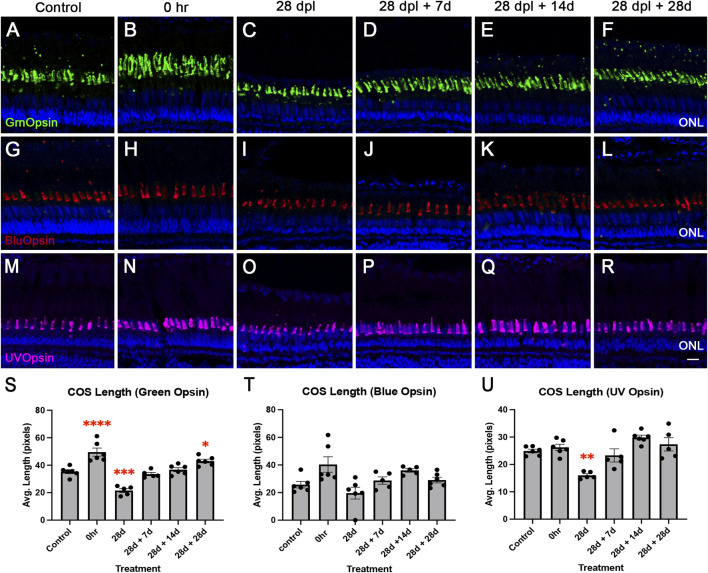
Gradual restoration in the cone outer segment (COS) was observed during a recovery period following CLL treatment. COS were immunolabeled with GrnOpsin [green; **(A–F)**], BlueOpsin [blue; **(G–L)**], and UVOpsin [pink; **(M–R)**]. TO-PRO-3 (blue) was used to stain nuclei. **(A,G,M)** control. **(B,H,N)** 0 h, **(C,I,O)** 28 dpl. **(D,J,P)** 28 dpl + 7 days of recovery. **(E,K,Q)** 28 dpl +14 days of recovery. **(F,L,R)** 28 dpl +28 days of recovery. **(S,T,U)** Graphical representations of quantitative data for COS. All comparisons were made to the control. **(S)** COS Green Opsin at 0 h exhibited a longer length than the control (p < 0.0001), with a 39% truncation observed by the end of CLL treatment (28 dpl; p < 0.0001). Following a return to normal light/dark conditions, COS length gradually recovered during the recovery period (*p = 0.0392). **(T)** COS Blue Opsin did not show any statistically significant differences in length across timepoints. **(U)** COS UV Opsin displayed a 36% reduction in length at 28 dpl (p = 0.0004), followed by gradual increases during recovery. N = 4–6 eyes analyzed per condition (values are mean ± SEM; scale bar in panel R = 15 µm).

Given that 28 days of CLL did not completely destroy rod and cone photoreceptors, we hypothesized that photoreceptor outer segments would recover to their normal length if CLL-treated fish were returned to normal light/dark conditions. Alternatively, it was possible that the chronic stress environment of CLL would prevent or delay recovery. To test these alternative possibilities, we collected retinas from untreated animals (control), after a 24-h dark adaptation (0 h), at the end of the 28-day CLL treatment (28 dpl), and at three points of recovery (28 dpl + 7d, 28 dpl +14d, and 28 dpl + 28d; Figure 6A). Next, we immunolabeled the collected retinas with Zpr-3 to label ROS (Figures 6B–G), PCNA to label proliferating cells (Figures 6H–M), and various cone opsins to label COS (Figures 7A–R). Following 28d CLL treatment, ROS showed significant truncation with 72% reduction compared to controls (p < 0.0001). This was followed by gradual increases in length during recovery, with near resolution after 28 days of recovery, where we observed that ROS length was only slightly shorter than controls (p = 0.0492; Figure 6N). Next, CellProfiler was used to quantify ONL rod nuclei. We found that by the end of CLL treatment (28d), ONL rod nuclei were reduced by 60% (p < 0.001) compared to control (Figure 6O). The number of nuclei gradually increased to 47% less (p < 0.001) by 7 days of recovery, 42% (p < 0.001) by 14 days of recovery, and 38% (p < 0.001) by 28 days of recovery compared to the control. As the ROS length recovered (Figure 6N), the number of PCNA + rod precursors in the ONL decreased (Figure 6P). Replicating previous findings (Kramer et al., 2023), we observed an approximately 10-fold (p < 0.0001) increase compared to baseline in PCNA + ONL nuclei at 28 d CLL (Figure 6P). This was followed by a gradual decrease at 7 days of recovery (p = 0.0007), 14 days of recovery (p = 0.0150), and resolution by 28 days of recovery (p = 0.6362; Figure 6O). A custom CellProfiler pipeline was used to validate these results and found similar trends (Figure 6Q). Finally, to characterize COS of red/green double cones, blue cones, and UV cones, we immunolabeled retinas from the same experiment with Green Opsin (green; Figures 7A–F), Blue Opsin (red; Figures 7G–L), and UV Opsin (pink; Supplementary Figure S5M–R), respectively. Here, we observed a differential susceptibility to CLL damage among the various cone subtypes. The COS labeled with Green Opsin and UV Opsin showed a significant truncation by 28 dpl, followed by gradual recovery (Figures 7S–U). However, we also found that blue cone photoreceptors were not as sensitive to CLL (Figure 7T), replicating our previous finding (Kramer et al., 2023). Collectively, these data suggest that CLL exposure induced significant but reversible structural damage to rod and cone outer segments, paired with a transient proliferation of rod precursor cells in the ONL. These changes ultimately largely restored retinal morphology to near-baseline levels after 28 days of recovery.

## 4 Discussion

Microglia, resident immune surveillance cells in the central nervous system (CNS), perform the crucial biological function of maintaining homeostasis in the retina and broader CNS (Fricker et al., 2012; Fu et al., 2014; Gogoleva et al., 2019; Nie et al., 2025; Paolicelli et al., 2011). They act as specialized macrophages, serving as the first line of phagocytotic defense through chemotactic motility to migrate in response to photoreceptor damage or dysfunction (Mitchell et al., 2018a; Nie et al., 2025). Additionally, microglia play an important role in the inflammatory response through a biphasic immunomodulatory mechanism. In response to a damaging stimulus, microglia can be activated into varying forms from M1 (pro-inflammatory) and M2 (anti-inflammatory) states (Rodríguez-Gómez et al., 2020). In particular, depending on environmental cues, they can secrete a mix of pro-(IL-1β, IL-6, and TNF-α) and anti-(IL-4, IL-10, and IL-13) inflammatory cytokines (Frank et al., 2007; Jesudasan et al., 2021; Lu and Hyde, 2024; Rodríguez-Gómez et al., 2020). However, although microglia are beneficial for maintaining homeostasis and facilitating retinal repair, they may also play a deleterious role in driving pathological progression of neurodegenerative disorders. For example, excessive and prolonged secretion of these neuromodulators by microglia leads to a cascade of neurotoxic effects (Nie et al., 2025). This indicates that an appropriate balance of pro- and anti-inflammatory cytokines is necessary for tissue maintenance and recovery from neuronal damage (Cicchese et al., 2018; Soliman and Barreda, 2022).

In elucidating the role of microglia in the context of chronic retinal damage, we found notable differences between Dex-mediated pharmacological inhibition and *irf8−/−* genetic manipulation. In particular, we observed significantly more damage to ROS/COS in Dex treatment (Figure 4). We propose that both Dex-mediated pharmacological inhibition and *irf8−/−* genetic manipulation inhibit normal microglial development but do so by interfering with opposing M1/M2 polarization states. In support of this possibility, a previous study found that high-dose dexamethasone resulted in neurotoxic effects and inhibition of M1 and M2 microglia, but with a greater inhibitory effect on M2 microglia both *in vitro* using BV2 cells and *in vivo* using a mouse model of traumatic brain injury (Yang et al., 2025). As M2 microglia release anti-inflammatory cytokines to promote damage resolution, reduced M2 polarization may result in exacerbated damage, as shown in our model of chronic degeneration (Figure 4).

In contrast to Dex treatment, the inhibition of microglia function in the *irf8−/−* line resulted in a partial protection from rod and cone damage during CLL (Figure 5). IRF8 is important for the direct induction of downstream M1-specific genes, including *IFN-β*, *IL-12*, and *iNOS* (Günthner and Anders, 2013; Xu et al., 2012; Zhou et al., 2018). In addition, in the rd1 mouse model of retinal degeneration, a lowered Irf8 expression attenuated neuroinflammation by shifting microglia polarization toward M2 (Zhou et al., 2018). We hypothesize that a similar inhibition of a pro-inflammatory state had a protective effect on photoreceptors in our *irf8−/−* model. Alternatively, it has also been shown that *irf8−/−* fish exhibit a disruption in normal microglia differentiation during early development (Shiau et al., 2015). This may result in chronic changes to retinal homeostasis and response to inflammation in adulthood. In support of this possibility, *irf8−/−* mutants exhibited increased MG gliosis, enhanced astrocyte activation, and cone photoreceptor loss in aged zebrafish (Song et al., 2024). Regardless of the underlying mechanism, our data suggest that Dex treatment and *irf8* null mutations result in differential outcomes on retinal homeostasis and response to neuronal damage.

We were surprised to find that the level of damage observed with Dex treatment did not result in MG proliferation, as we hypothesized. We propose two potential reasons for this finding. First, it is possible that Dex—because it is a glucocorticoid—had off-target effects on MG or metabolic stress-related processes. In support of this possibility, other studies using glucocorticoids noted effects on retinal stem cells that were unrelated to microglial inhibition (Emmerich et al., 2023; Gallina et al., 2014; Grisé et al., 2021). A second possibility stems from our finding that despite greater damage to the COS, cone cell somas remained intact (Figures 4I–L). Previous research has suggested that cone cell death may be required to trigger MG proliferation (Lenkowski and Raymond, 2014). This is supported by differential responses observed in two genetic mutations: a XOPS-mCFP transgenic line that resulted in rod photoreceptor degeneration and a *pde6c* null mutation that resulted in cone photoreceptor degeneration (Morris et al., 2008; Morris et al., 2005). The XOPS-mCFP line showed proliferation of rod precursors in the ONL—similar to our observations in the CLL model (Kramer et al., 2023; Turkalj et al., 2021)—but exhibited no MG proliferation (Morris et al., 2005). In contrast, the *pde6c* line showed minimal rod precursor proliferation, but there was MG cell-cycle re-entry (Morris et al., 2008).

Previous findings showed partial restoration of ROS/COS after 10 days of the CLL protocol and a 2-week recovery (Kramer et al., 2023). In this study, we found that although full MG proliferation was not triggered after a 28-day CLL time course, after 28 days of recovery, the retinal photoreceptors exhibited a restoration of gross structural morphology (Figure 7). We hypothesize that rod recovery was likely due to a combination of rod precursor generation of new rods and subsequent ROS outgrowth. This is supported by our finding that PCNA + rod precursors decreased over the recovery process (Figure 6P) and that there was a statistically significant increase in the number of rod nuclei from the end of treatment (28d) to 28 days of recovery (28d + 28d) (Figure 6O, p < 0.05). Similarly, cone recovery was likely due to the regeneration of the COS as photoreceptor outer segments undergo continuous renewal to ensure sustained health (Xu et al., 2024). However, it remains unclear whether the observed kinetics of ROS and COS recovery following CLL is at the rate of normal outgrowth. Regardless, these data imply that even with prolonged sublethal stress, zebrafish retained the capacity for OS repair. This is particularly important for understanding how disruptions in OS renewal contribute to the progression of genetic disorders such as retinitis pigmentosa (Xu et al., 2024).

Although we found that 28-day recovery following CLL led to structural photoreceptor recovery, we did not test whether structural recovery translated into functional recovery. We acknowledge this as a limitation and aim to incorporate behavioral tests in future experiments to characterize visual performance. In a neurotoxic model of whole retinal destruction, full regeneration and functional recovery required 100 days in cases of severe retina damage (Sherpa et al., 2008; Sherpa et al., 2014). Another group found that retinal regeneration following AL damage resulted in a gradual restoration of contrast sensitivity, special resolution, and color perception (Hammer et al., 2022). This is notable as nearly all rod and cone photoreceptors were destroyed and must be replaced following acute light damage (Kramer et al., 2023). In contrast, in the CLL model, only ∼50% of rod photoreceptors are lost (Kramer et al., 2023); following recovery, newly formed rods must rewire only the lost connections within the context of a partially established neuronal network. It remains unclear whether this process is “easier” or “more difficult” than the full rewiring needed to recover from AL damage. Characterizing the mechanism that underlies potential vision restoration in the CLL model may provide insights into the feasibility of improving vision through stem cell implantation in cases of partial retinal degeneration. Finally, as we only tracked animals for 28 days of recovery, future work should assess later time points to characterize long-term stability of the network. These experiments could assess whether appropriate synaptic reconnections are maintained beyond this point, and if not, whether newly formed rod photoreceptors undergo subsequent degeneration and limit functional recovery.

We also acknowledge that the CLL paradigm represents a simplified model of retinal neurodegeneration and does not fully recapitulate the complex pathology observed in human conditions such as diabetic retinopathy or age-related macular degeneration. Future studies using models that incorporate vascular and metabolic dysfunction will be important to validate the broader translational relevance of these findings. In addition to this, the present analysis mostly focused on transcriptional changes through a microglia-specific gene expression analysis. Based on previous work (Kramer et al., 2023), we also conducted a simple analysis of the microglia area in the ROS and found that there was no difference in morphology upon Dex treatment (Figure 3F). As retinal microglia display distinct morphologies that are associated with various states of activation, future efforts may include a more comprehensive classification of changes in morphology. Currently, it is difficult to fully capture the nuanced morphology of microglia due to the limitations of using thin section immunohistochemistry. However, new tools are emerging to potentially overcome this limitation. One example is CellProfiler, free and open-source image analysis software used in this study for unbiased object identification (Carpenter et al., 2006). Although we have previously quantified zebrafish retinal photoreceptors following chronic low light injury (Berry et al., 2025), future efforts may include integrating morphology-based microglial characterization with a machine learning-based classification algorithm. Others in the field have also begun to develop more advanced and nuanced analyses of microglia, creating the first tool to perform skeletonization and arborization areas of ramified microglia branches (Sánchez-Puebla et al., 2025). However, these studies were performed with flat-mounted retinas, which also have their own limitations regarding depth of field.

Our findings highlight the challenges of integrating genetic, morphological, and functional analyses to fully understand the role of microglia in retinal degeneration and repair. Some investigators have recognized this and have incorporated macrophages into retinal organoid co-cultures to better understand the role of microglia in retinal diseases and in identifying more effective therapies (Usui-Ouchi et al., 2023). Notably, our findings demonstrate that both pharmacological and genetic approaches to microglial modulation in retinal disease can result in either neurotoxic or neuroprotective outcomes, depending on how they influence microglial function. From a clinical perspective, this suggests that selective modulation of microglial polarization, particularly promoting a shift from the pro-inflammatory M1 to an anti-inflammatory M2 phenotype, may be more favorable than global microglial suppression. For example, in the treatment of diabetic retinopathy, emerging therapies are targeting microglial polarization. A recent study reported that intravitreally injected nanoparticles carrying rapamycin in a mouse model of DR effectively promoted microglial transition to an M2 anti-inflammatory state, resulting in amelioration of diabetic retinopathy (Li et al., 2025; Niu et al., 2025).

Overall, our results underscore the complex role of microglia in chronic retinal injury. Delaying or preventing vision loss in chronic retinal degeneration may ultimately depend on a deeper understanding of how microglia regulate neuroinflammation and promote neuroprotection.

## Data Availability

The datasets presented in this study can be found in online repositories. The names of the repository/repositories and accession number(s) can be found below: https://www.ncbi.nlm.nih.gov/, GSE233896.
