# Supplementary material for: Microglia response and function in a chronic model of photoreceptor damage
Source: Front Cell Dev Biol. 2025 Dec 16;13:1699271. doi: 10.3389/fcell.2025.1699271 (PMC12748171; doi:10.3389/fcell.2025.1699271)
Supplement: Supplementary file 1 [file DataSheet1.docx]

Supplementary Material


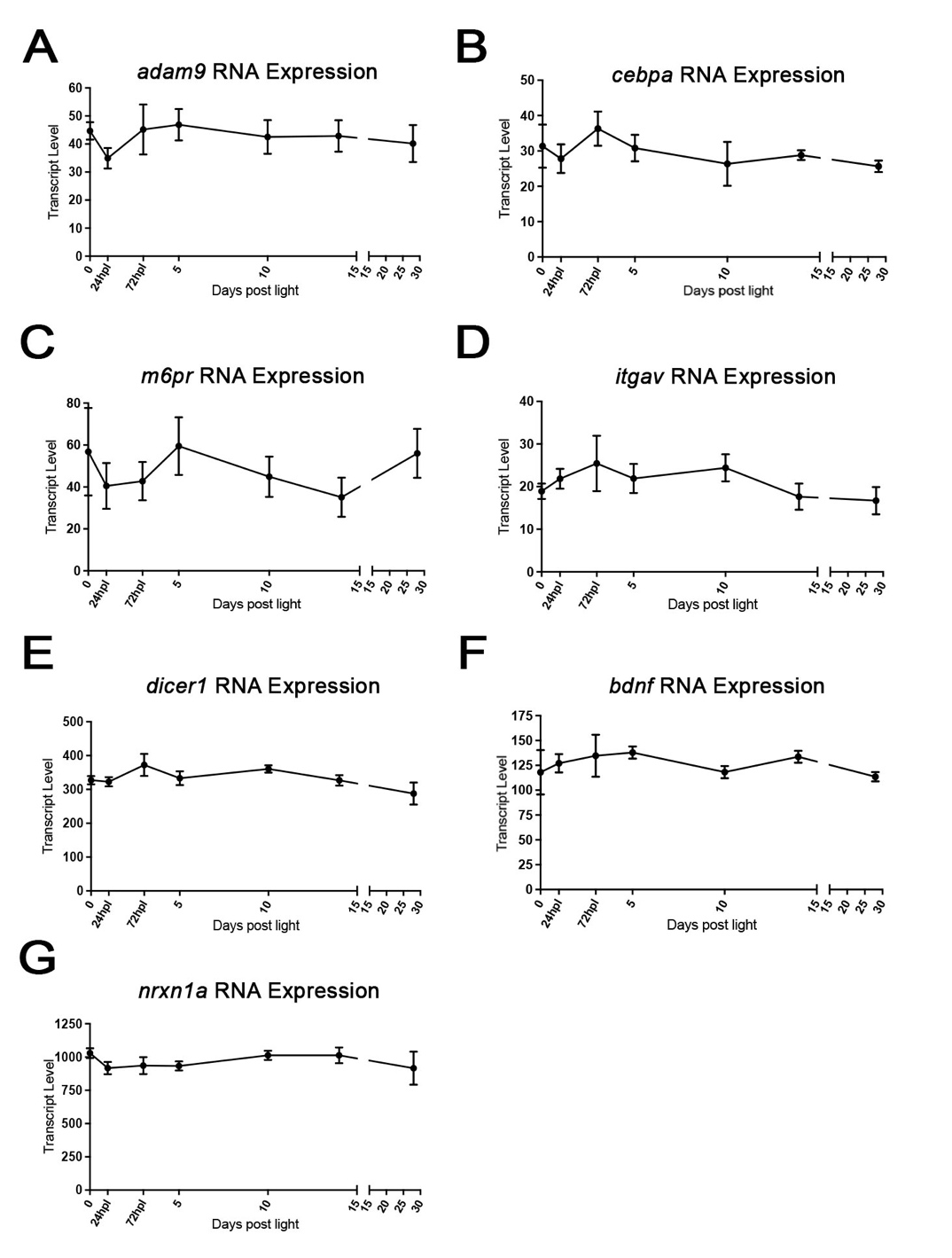
**Supplementary Fig. 1. Temporal expression profiles of microglia-associated genes with stable expression during the CLL response.**Transcript levels were analyzed across seven time points following CLL damage: 0 hr, 24 hpl, 72 hpl, 5 dpl, 10 dpl, 14 dpl, and 28 dpl.
(**A–G**) Expression levels of *adam9*, *cebpa*, *m6pr, itgav, dicer1, bdnf, nrxn1a* showing stable expression. Error bars indicate SEM.


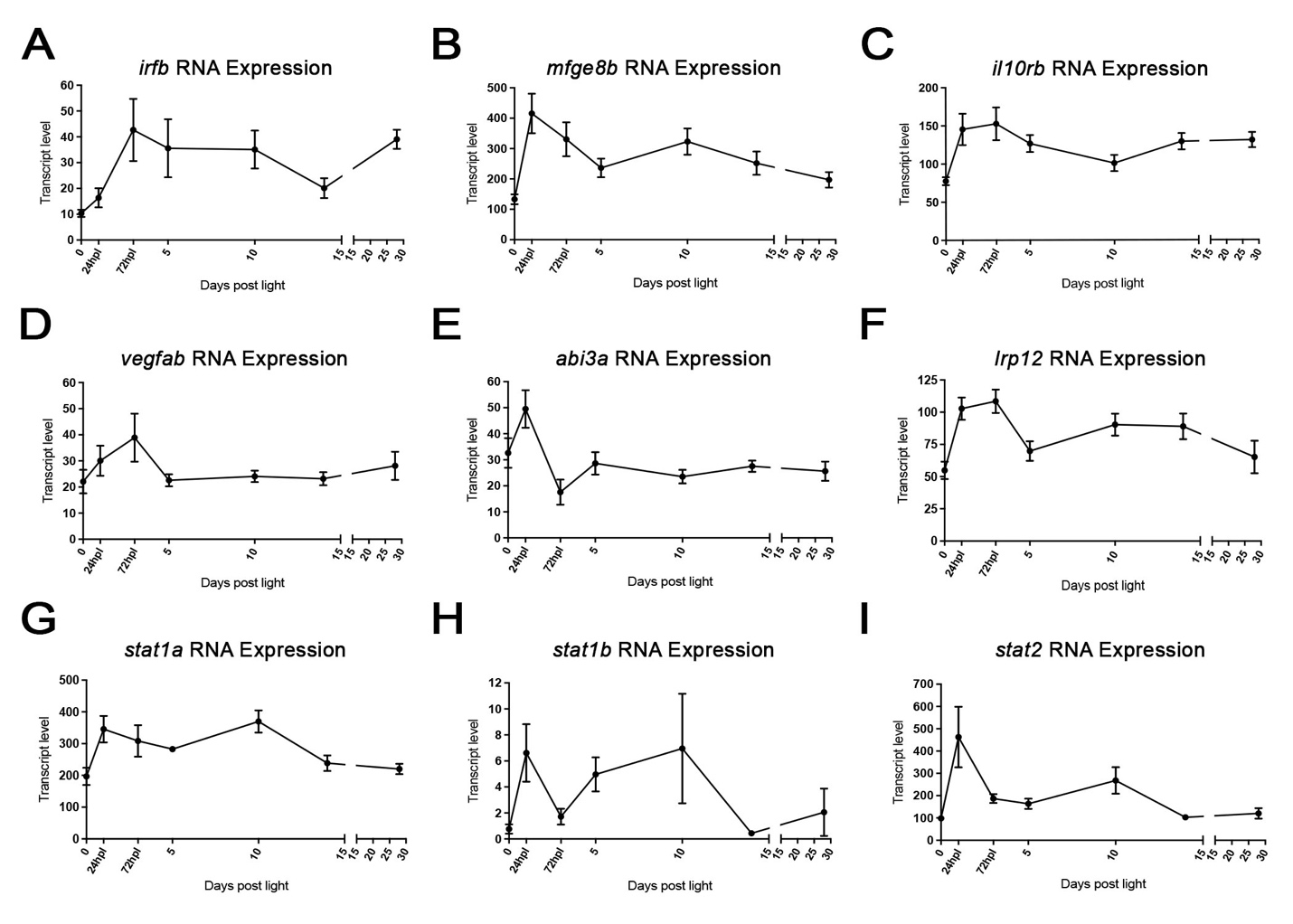


**Supplementary Fig. 2. Temporal expression profiles of 9 microglia-associated genes with an upregulation early in the CLL time course.** Transcript levels for genes retained in the final dataset were analyzed across 7 time points following CLL damage: 0 hr, 24 hpl, 72 hpl, 5 dpl, 10 dpl, 14 dpl, and 28 dpl. (**A - I**) *irfb*, *mfge8b*, *il10rb*, *vegfab*, *abi3a*, *lrp12*, *stat1a*, *stat1b*, and *stat2* all showed an early rise and peak trend between 24 hpl and 72 hpl across the CLL time course. Error bars indicate SEM.

**
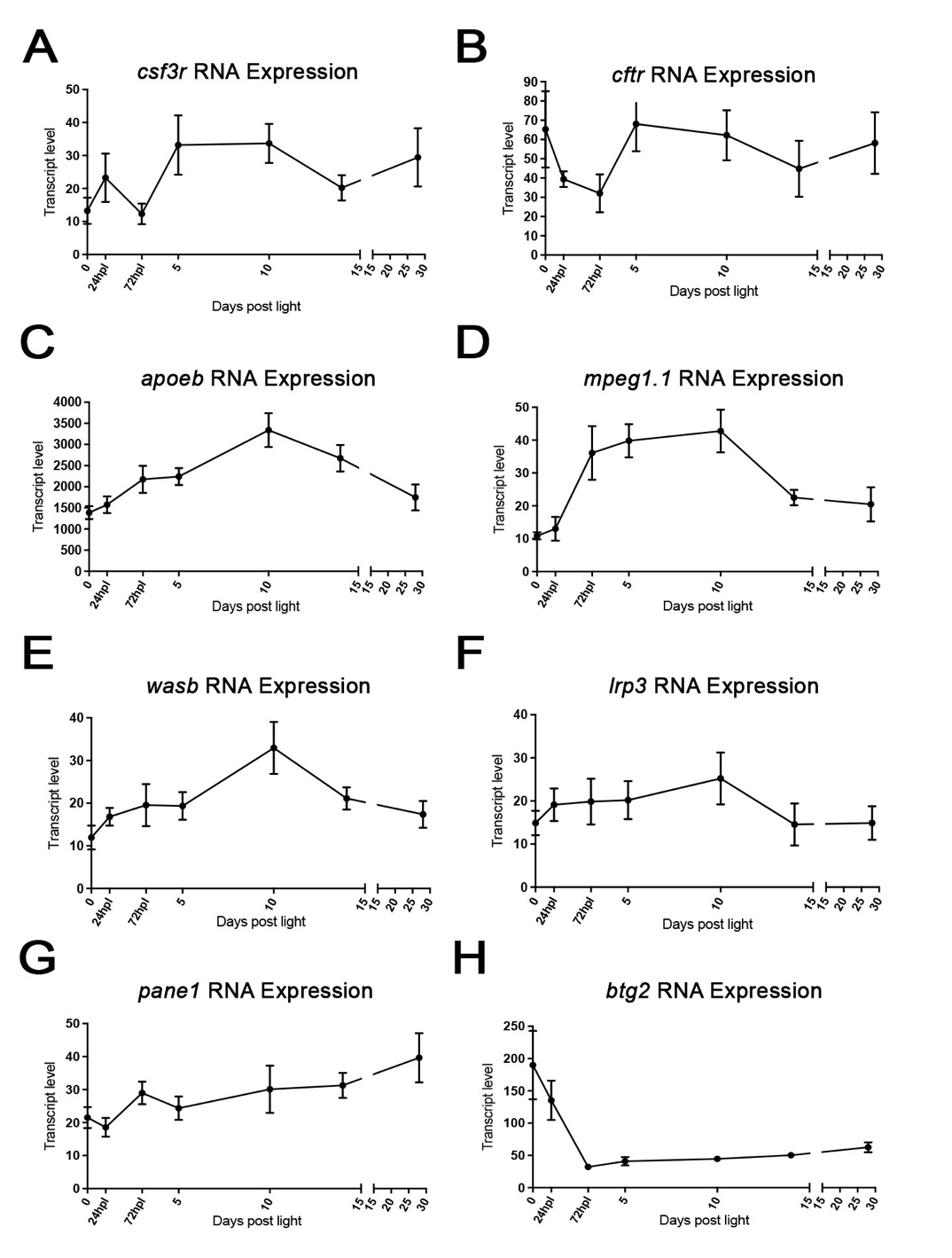
Supplementary Fig. 3. Temporal expression profiles of 8 additional microglia-associated genes revealed 4 more regulatory patterns during the CLL response.**Transcript levels were analyzed across seven time points following CLL damage: 0 hr, 24 hpl, 72 hpl, 5 dpl, 10 dpl, 14 dpl, and 28 dpl. (**A–B**) Expression of *csf3r*, and *cftr* showed a peak at 5 dpl. (**C-F**) *apoeb*, *mpeg1.1*, *wasb*, and *lrp3* showed a peak at 10 dpl. (G)  *pane1* showed a peak at 28 dpl. (H) *btg2* showed a peak at 0 hr, and then was downregulated throughout the time course. Error bars indicate SEM.

**
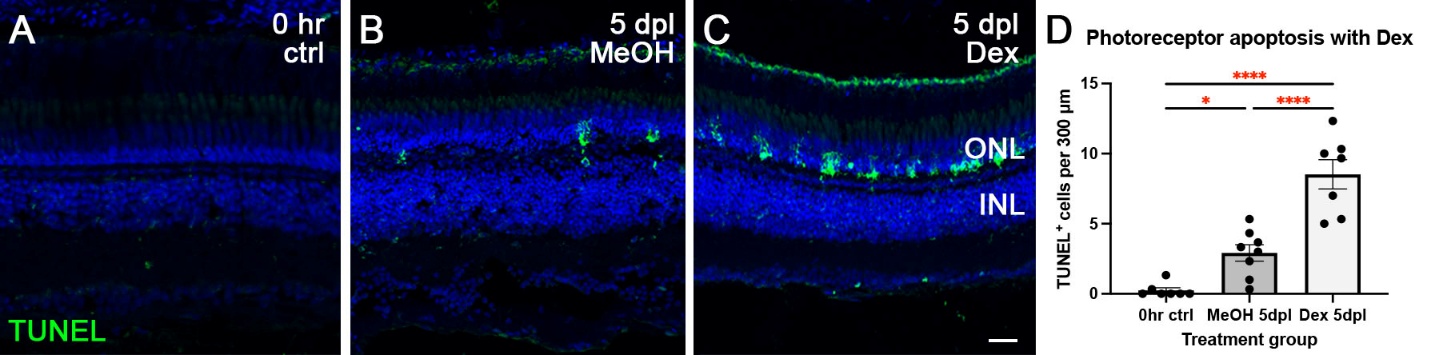
**

**Supplementary Fig. 4. Photoreceptor apoptosis was exacerbated with Dex treatment.** Control (**A)**, MeOH 5dpl (**B**), and Dex-treated 5dpl (**C**) retinas stained with TUNEL (green) to label photoreceptor apoptosis. TO-PRO-3 (blue) was used to stain nuclei. (**D**) Compared with controls, there was a 12-fold (p=0.321) increase in photoreceptor apoptosis with MeOH, and 36-fold (p<0.0001) increase with Dex treatment as compared with controls. N=6-8 eyes analyzed per condition (values are mean ± SEM; scale bar in panel C=15 µm).
